# Integration of Underwater Radioactivity and Acoustic Sensors into an Open Sea Near Real-Time Multi-Parametric Observation System

**DOI:** 10.3390/s18082737

**Published:** 2018-08-20

**Authors:** Sara Pensieri, Dionisis Patiris, Stylianos Alexakis, Marios N. Anagnostou, Aristides Prospathopoulos, Christos Tsabaris, Roberto Bozzano

**Affiliations:** 1 Institute of Marine Science, National Research Council of Italy, Via de Marini 6, 16149 Genova, Italy; roberto.bozzano@cnr.it; 2 Institute of Oceanography, Hellenic Center for Marine Research, 19013 Anavyssos, Greece; dpatiris@hcmr.gr (D.P.); salexakis@hcmr.gr (S.A.); aprosp@hcmr.gr (A.P.); tsabaris@hcmr.gr (C.T.); 3 National Observatory of Athens, IERSD, 15236 Athens, Greece; sifneos@live.com; 4 Department of Water Resources, School of Civil Engineering, NTUA, 10682 Athens, Greece; 5 HoRST S.P. Private Company, Alepou 49100 Corfu, Greece; info@horst.gr

**Keywords:** rainfall, marine technology, underwater spectroscopy, passive acoustic

## Abstract

This work deals with the installation of two smart in-situ sensors (for underwater radioactivity and underwater sound monitoring) on the Western 1-Mediterranean Moored Multisensor Array (W1-M3A) ocean observing system that is equipped with all appropriate modules for continuous, long-term and real-time operation. All necessary tasks for their integration are described such as, the upgrade of the sensors for interoperable and power-efficient operation, the conversion of data in homogeneous and standard format, the automated pre-process of the raw data, the real-time integration of data and metadata (related to data processing and calibration procedure) into the controller of the observing system, the test and debugging of the developed algorithms in the laboratory, and the obtained quality-controlled data. The integration allowed the transmission of the acquired data in near-real time along with a complete set of typical ocean and atmospheric parameters. Preliminary analysis of the data is presented, providing qualitative information during rainfall periods, and combine gamma-ray detection rates with passive acoustic data. The analysis exhibits a satisfactory identification of rainfall events by both sensors according to the estimates obtained by the rain gauge operating on the observatory and the remote observations collected by meteorological radars.

## 1. Introduction

A comprehensive understanding of the processes and conditions that affect the state of the marine environment is a key parameter in many fields of environmental science and engineering as well as in natural hazard forecasting efforts. Climate conditions and variations, floods and prolonged seasons of high temperatures and droughts, water pollution prevention and sustainable management of marine resources, renewable ocean energy and maritime transport are topics of high social and scientific impact that relies on deep understanding of the oceans functionality. This understanding is strongly dependent on observations and monitoring of key parameters at a wide range of space and time scales. For this purpose, technologically advanced solutions are steadily emerging such as stationary and mobile observing platforms, new sensors and auxiliary communication modules for smart and real-time monitoring of essential seawater parameters.

The increase of technological and engineering efforts towards the integration of innovative sensors into existing multi-parametric observing systems was also favored by the Marine Strategy Framework Directive (MSFD) [1] that introduced new parameters to describe the Good Environmental Status of the seawater. Specifically, human-induced marine underwater noise was defined a pollutant and its monitoring was considered essential to assess its effects on marine life; similar attention was paid to increase the monitoring of chemical and radioactive elements and compounds that potentially could be contaminants.

Until now, existing acoustic surveys have been typically oriented to study sea mammal’s behavior and protection, although underwater sounds could be of interest to monitor rainfall and wind over the sea surface as well as shipping noise. Few observatories, mostly cabled, have been equipped with hydrophones to listen to the ocean in a continuous way (e.g., [2,3,4]), but several experiments of limited duration have been carried out using ARGO floats [5] or fixed stations [6,7].

On the other hand, the use of autonomous devices able to monitor chemical compounds is still missing due to the considerable power requirements to perform measurements based on analytical methods. Some steps forward have been made as regards the monitoring of radioactivity through calibrated measurements for studying oceanic processes [8]. In the marine environment, in situ measurements of radon progenies and potassium (^40^K) together with underwater sound records have been utilized as tracers of submarine groundwater discharge [9] and rainfall in the open sea [10]. Also plenty of measurements of artificial radioactivity have been occurred after the Chernobyl accident [11] for radiological surveillance and monitoring purposes.

Although autonomous sensors able to monitor underwater sound and marine radioactivity can be complementary each other to study rainfall or to detect groundwater discharges, their integration into oceanic platforms is not so conventional due to the intrinsic difference of their operating mode, quality control and quality assurance procedures, heterogeneity of data stream, data processing algorithms, and last but not least due to the power required for continuous and long-term operation. In this frame, the main technical challenges consist in: (a) downsizing the sensors, (b) increasing the channels of serial outputs function of the electronics onboard the observing systems that have the role to schedule the sampling scheme and (c) guaranteeing enough power supply to the scientific payload to be continuously operational. 

Among the platforms operational at sea, the Eulerian observatories are the most useful to integrate such kind of instruments, since they allow measurements with a multi-annual temporal scale of operation also in harsh environments (i.e., deep sea, polar regions, highly trafficked basins, etc.) [12]. Fixed observatories have also the capability to fulfill the concept of the Essential Ocean Climate Variables [13] collecting a variety of parameters spanning from the marine physics to bio-geo-chemistry, from meteorology to sea state and water quality that are ancillary to acoustic and radioactivity measurements.

In the last decades, the creation, development and management of fixed-point coastal and offshore observatories for marine research was mainly sustained by a great variety of projects funded by the European Commission or the Member States. Several European (i.e., EuroSITES, JERICO, FIXO^3^). In addition, national networks (i.e., IFON, POSEIDON, MOOSE, IEOOS, MARNET) were developed to deepen the knowledge about the trends of ocean physics and biochemistry and a huge effort has been concerted to integrate part of them into worldwide open ocean observatories initiatives such as OceanSITES [14].

More recently, a new form of integration and harmonization was put beside the traditional research-oriented support scheme based on the community legal framework for a European Research Infrastructure Consortium (ERIC). The fixed-point marine observatories are a fundamental component of some initiatives within the ERIC frame, such as the Integrated Carbon Observation System (ICOS) and the European Multidisciplinary Seafloor Observatory (EMSO).

In this international frame, the integration of a radioactivity spectrometer (KATERINA II) and an underwater passive aquatic listener (UPAL) into the fixed-point oceanic observatory Western 1-Mediterranean Moored Multisensor Array (W1-M3A) in the North Western Mediterranean Sea was carried out as first attempt to simultaneously use radioactivity and underwater acoustic data for near-real time monitoring rainfall in the open sea.

Section 2 describes the area of the experiment and the characteristics of the marine observatory used to host the two sensors. Section 3 and Section 4 detail the new features of the sensors and their integration into the W1-M3A monitoring system. Then, preliminary results using the collected data are presented in Section 5, and concluding remarks are laid out in Section 6.

## 2. Deployment Sites and Existing Infrastructure

### 2.1. Deployment Site

**Figure 1:**
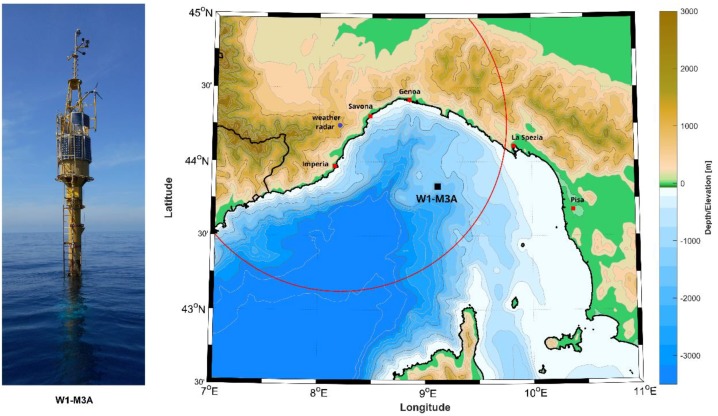
The surface buoy of the W1-M3A observing system and the map of the Ligurian basin. The square at the center of the basin marks the position of the W1-M3A observing system, the circle shows the operational range of the weather radars in the Liguria region used in the analysis.

The area of the experiment is the Ligurian Sea, a semi-enclosed basin in the North-West Mediterranean Sea (Figure 1). The basin is characterized by a complex orography, since it is surrounded on the North by Liguria region with steep mountains close to the coasts and on the South by the Corsica Island. These constraints render the basin an ideal site for studying air-sea interactions [15] and dense water formation, that occur especially in winter [16]. The area is also characterized by cyclogenesis and self-regenerating thunderstorms which mainly cause the frequently occurred floods on the coasts of Liguria and Tuscany Regions in the recent years [17,18].

### 2.2. The W1-M3A Observing System

The W1-M3A observing system [19] is part of the M3A network of permanent observatories established in the 90 s to collect data on long term physical and meteorological parameters in open sea [20] for the benefit of the Mediterranean ocean forecasting system [21]. It also belongs to OceanSITES and to the Italian network of fixed open ocean observatories known as IFON [22].

There are two main installations that form the W1-M3A marine observing system: a spar surface buoy permanently moored on a 1200 m seabed, and a separate subsurface mooring, deployed about 4 km far from the main installation to avoid entaglement with the mooring-line of the surface buoy. The buoy is still one of the few examples of spar platforms operating in the Mediterranean Sea, designed to maintain stability even in rough seas and high waves and allowing scientists to make very precise measurements [23].

The surface buoy is equipped with GPS, camera, a complete set of stand-alone meteorological sensors (barometer, 2D and 3D sonic anemometer, thermo-hygrometer, pyranometer, pyrgeometer, par, CO_2_/H_2_O analyzer) and a compact weather station providing measurements of wind speed and direction, air temperature, relative humidity, atmospheric pressure and rainfall. The latter parameter is measured using the RAINCAP sensor that infers the cumulate by the acoustic impact of drops on the sensing element [24]. This instrument was recently used for its reliability to monitor rainfall on both land and at sea [25,26,27].

The submerged part of the W1-M3A observatory is equipped with conductivity-temperature-depth (CTD) devices, biochemical sensors (for monitoring dissolved oxygen, chlorophyll-a, turbidity, nutrient, pCO_2_ and pH), and properly positioned echo-sounders to estimate wave characteristics (significant wave height, period and main direction of propagation). The subsurface mooring housed CTDs at different depths from the near surface to the ocean interior for monitoring the physics of the water column.

**Figure 2:**
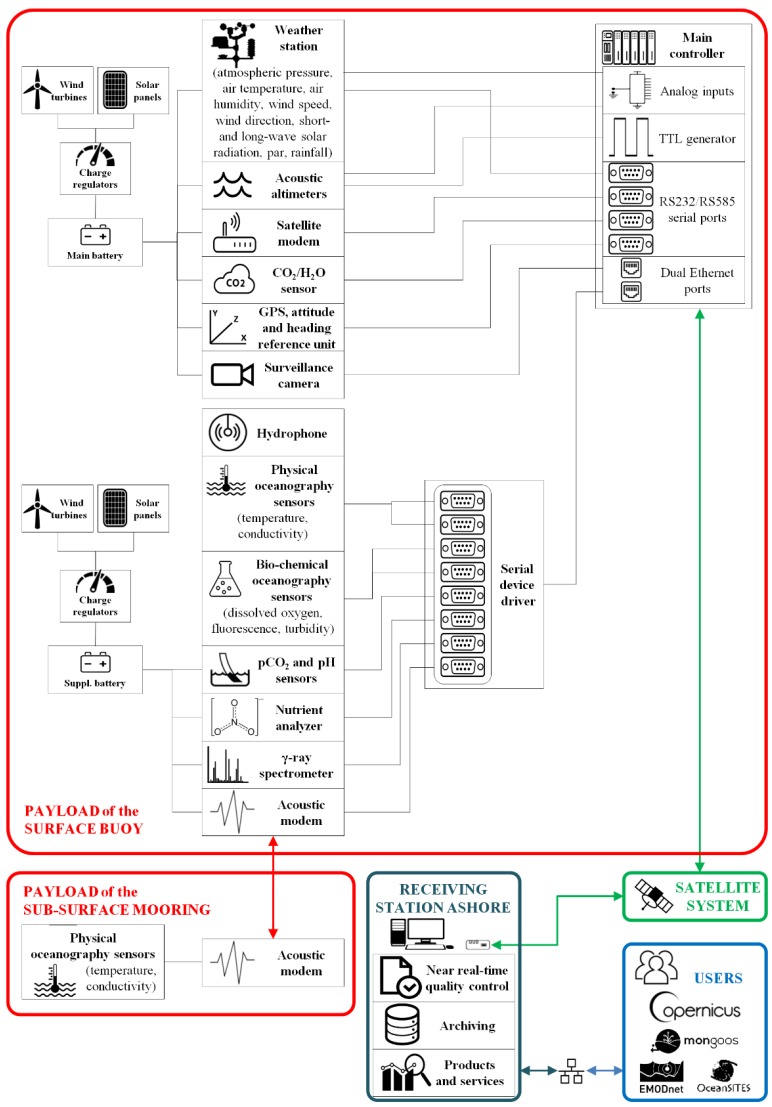
Scheme of the main components constituting the W1-M3A observatory.

The architecture of the W1-M3A observatory, consisting mainly of the scientific payloads, the data acquisition-transmission system and the receiving station ashore, is depicted in Figure 2. The system devoted to the acquisition of signals is based on a real-time controller that collects the measurements provided by all instrumentation onboard the buoy and merges them in compressed data files, that are stored in the internal memory, on an hourly basis. The full time series of the meteorological parameters is processed on board to obtain statistical quantities such as average, standard deviation and range of variation, whereas the three-time series of the echo-sounders, properly corrected for buoy’s motion [28], are processed to get estimates of significant wave height, period and main direction of propagation of the ocean waves.

Statistical values with oceanographic measurements are merged, stored in the on board memory and transmitted in near-real time to the station ashore by means of an IRIDIUM satellite link. The tasks of the receiving station are to: (a) automatically decode the received data files, (b) apply quality control procedures compliant with the Data Buoy Cooperation Panel (DBCP) Quality Control Guidelines, and (c) archive the data on a local MySQL relational database. Automatic routines have also been developed to format some subset of data in the OceanSITES NetCDF standard and in the Binary Universal Form for the Representation of meteorological data (BUFR) of the World Meteorological Organization. These coded data are uploaded to international repositories such as the data center of the Mediterranean Operational Network for the Global Ocean Observing System (MonGOOS), the Marine Service of Copernicus, the European Marine Observation and Data network (EMODnet) physics portal and OceanSITES through the CORIOLIS Global Data Assembly Center (GDAC).

## 3. Sensor Upgrade

### 3.1. The KATERINA II Underwater Radioactivity Sensor 

KATERINA II is a compact and autonomous underwater gamma-ray spectrometer capable of monitoring radon ^222^Rn and thoron ^220^Rn progenies (^214^Bi, ^214^Pb, ^208^Tl) and radioactive potassium ^40^K. For the detection of gamma-rays, a scintillation crystal of NaI(Tl) (7.62 × 7.62 cm^2^) is used providing spectra of gamma-rays in a wide range of energy (50–2800 keV) based on a multi-channel analyzer of 1024 channels and other auxiliary electronic and memory modules. The NaI(Tl) crystal is ideal for prolonged application in harsh environmental conditions as it is solid and durable, needs no cooling or extremely high voltage input (5–18 V DC), and it can continuously operate for years without significant deterioration of its lattice. On the other hand, the resolution of the provided spectra is low with a relative full width at half maximum of 6.5% for ^137^Cs detection at the energy of 661 keV. The dead time (time needed for data processing) is less than 0.5% when the system operates in aquatic environment and the lower limit of detectability is around 20 Bqm^−3^ for an acquisition time of 24 h. All parts of the system are tightly assembled inside a cylindrical housing made of acetal, allowing maximum operation depth of 600 m. Acetal is a light and durable material which weakly interact with gamma-rays ensuring minimum gamma-ray attenuation during operation. 

The KATERINA II system is capable of easy integration into measuring platforms (both stationary and mobile). For its integration into the W1-M3A observing system the following improvements were implemented prior to the installation: (a) spectrum stability, (b) low power consumption and (c) modern communication protocols. 

A standard drawback of scintillation-based gamma-ray spectrometers is the spectrum drift during their operation due to temperature and voltage variations or other external conditions. With respect to previous versions, the spectrum variation due to voltage drifts was eliminated in KATERINA II, using digital electronics in the electronic modules to adjust the voltage output of the intense peak of ^40^K (at 1461 keV as measured due to the seawater constituents) in the middle of the spectrum (around the 512 channel). Also, due to slow and slight variations of temperature in the marine environment, the phenomenon is further reduced. As result, the stability of the acquired spectra (preset acquisition time of 1 h) allows to set regions of interest (ROI) in specific energy windows in areas of photo-peaks of interest and observe variations of the corresponding counting rates during the day. Also, to improve the precision of the measurements, several 1-h spectra can be summed and a spectrum corresponding to time intervals of 6, 12 or 24 h can be created in the post-process phase of data manipulation. The spectrum stability also ensures the quality of the results obtained by the automated spectrum analysis routines which were embedded in the controller onboard the buoy, providing quantitative results of radon ^222^Rn progenies at the gamma-ray energy of 351 keV (^214^Pb), 609 and 1764 keV (^214^Bi).

An important upgrade of the KATERINA II system with respect to previous versions concerns the drastic reduction in power consumption and the new communication protocol which allows easy data transfer and two-way communication. The electronic modules responsible for the power supply of the system were re-implemented allowing a power consumption lower than 1 W making possible its integration into the observation system and its continuous operation for periods of several months. Also, the upgraded communication protocol allows the data transfer via USB, RS232 and/or Ethernet protocols. Appropriate software was developed providing two-way communication with any operational data-logging system. For the needs of the current deployment, the data-logger of the system was adequately programmed to operate via RS232 interface. The data-logger consist of a compact stand-alone digital Multi-Channel Analyzer with a fully controlled micro-processor to perform data acquisition using digital signal processing algorithms and other functionalities for communication with other sensors in the same monitoring platform. Furthermore, software functions were also developed to achieve communication with the external controller of the observing system. In the laboratory, this was done at different levels using commands in hexadecimal format and performing all steps of communications with the system (via RS232 communication protocol). In order to achieve an effective communication between the sensor and the computer station, the desirable option is to send the commands individually. The second step consisted of the following tasks: (a) a dedicated program is developed to program the flow of the individual commands, and (b) the quality control of commands are validated from the response of the system as an acknowledgement receipt.

The KATERINA II detection system as installed in the W1-M3A observing system, provides three different data sets with a time-lag (acquisition time) of 1-h: real-time data of total gamma-ray rate (gross counts per second), real-time qualitative data of ^222,220^Rn progenies and ^40^K (counts per second in ROIs corresponded with each radionuclide without background subtraction) and post automated quantitative activity concentration of ^222,220^Rn progenies and ^40^K (background subtraction and efficiency consideration for each photo-peak). The latter is accomplished after the retrieving of the whole energy spectra from the internal memory during maintenance operation. The counting rates of specific photo-peaks, after the subtraction of the Compton background, are converted into absolute units of Bq l^−1^ or Bq m^−3^ using the photo-peak detection efficiency, which is an energy-depended parameter and its estimation is described elsewhere [29].

### 3.2. The UPAL Underwater Passive Aquatic Listener 

UPAL is an autonomous innovative instrument specifically conceived to collect and process underwater ambient noise data in real-time on any ocean mooring. A real-time embedded operating and signal processing software runs on its microprocessor to detect and to classify sources into geophysical (wind speed, rain rates and type, sea roughness), anthropogenic (ships, sonars, etc.) and biological (marine mammals, etc.). Quantitative estimation is also provided for wind and rain [6].

The instrument is based on cutting-edge hardware and software engineering technologies consisting of a high-sensitive low-power broadband hydrophone with linear working frequencies down to 5 Hz and up to 90 kHz [30]. The nominal sensitivity of the used hydrophone is −160 to −155 dB relative to 1 V/µPa and the equivalent oceanic background noise level of the pre-amplifier system is about 28 dB relative to 1 µPa^2^Hz^−1^. The intrinsic noise of the frequency response is greater than 10 dB below Sea State “0” (dB rel. 1 µPa/√Hz) and for the used frequencies the values are 21.6, 17.3, 15.5, 13.3, 12.4, 11.3, 10.5 dB rel. 1 µPa/√Hz at the 2, 5, 8, 15, 20, 30, 40 kHz, respectively.

With respect to previous versions, the system has new low-consumption active filters with better cut-off frequencies, high internal data storage components (up to 128 Gb), higher capacitance batteries (>65 Ah) and the versatility to be connected to any external power station. The innovative design is based on new low power, low noise, high bandwidth, wide dynamic range combined microcomputer/microprocessor and analogue-to-digital converter (ADC), based on Field Programmable Gate Array (FPGA) technology capable of accommodating the firmware and the smart processing algorithm.

One of the most innovative features of UPAL is its capability to autonomously adapt the duty cycle (sleeping and listening periods) depending on the source of the acoustic signal and mission requirements. When active, UPAL performs two tasks: the first is devoted to data collection for a specified time interval during which the instrument samples the audio output of the hydrophone at 100 kHz for a time slot of 4.5 s, and performs a Fast Fourier Transform (FFT) using the onboard FPGA to obtain a frequency spectrum of 512 sound pressure levels (SPL) over the entire frequency range of 0.5–50 kHz. The second task averages over a 1 kHz bandwidth eight discrete frequencies (1, 2, 5, 8, 15, 20, 30, and 40 kHz) of the spectrum and applies a tree model approach using these SPLs to classify the acoustic source. Each different detected source is associated to a flag (a specific code for each detected sources of the noise). The basic assumption is that geophysical generated sounds from rain, drizzle or wind are generally stationary even over a short time interval, whereas banging from ships or moorings, or chirps, whistles or clicks are sound signals that usually are non-stationary over a certain time interval.

The aforementioned evaluation process is used to set the listening (wake up/sleeping) modes of the system saving energy between data collection sequences. This makes the device suitable also to be used as trigger for several monitoring applications such as the groundwater emission from the sea bottom [9].

Once the noise source is identified as wind or rain, the SPLs at specific frequencies can be used to estimate wind speed or rain rate, respectively [6]. At the end of the duty cycle, the system creates a “message” including the values of the SPLs used for the classification, the flags and the quantitative estimates resultant from the classification and quantification algorithms, respectively. The message is made available to any external data logger through RS232 or USB interfaces for near real-time transmission ashore.

## 4. Sensors Integration into the W1-M3A Monitoring System

The software embedded in the controller of the W1-M3A observatory has been fully developed by the research group of the National Research Council of Italy who takes care of the technological and scientific development of the platform in such a way that innovative sensors can be integrated straightforward into the platform after all needed software and hardware interfaces have been developed.

As soon as the executable program starts, the controller gets several parameters of configuration from an initialization file stored locally: the calibration coefficients of the sensors, the duration of the duty cycle, the scheduled start-up time or period for each instruments or group of homogenous of sensors, the sampling frequency for each input analogue channel and the parameters related to the data transmission (i.e., frequency of outgoing call, allowed phone numbers of the receiving station ashore, maximum number of files that can be transmitted for each call, etc.). Once the configuration phase is completed, the program operates uninterruptedly.

Although each mentioned parameter can be configured by users for special purposes, the normal operating mode consists of a duty cycle of one hour.

**Figure 3:**
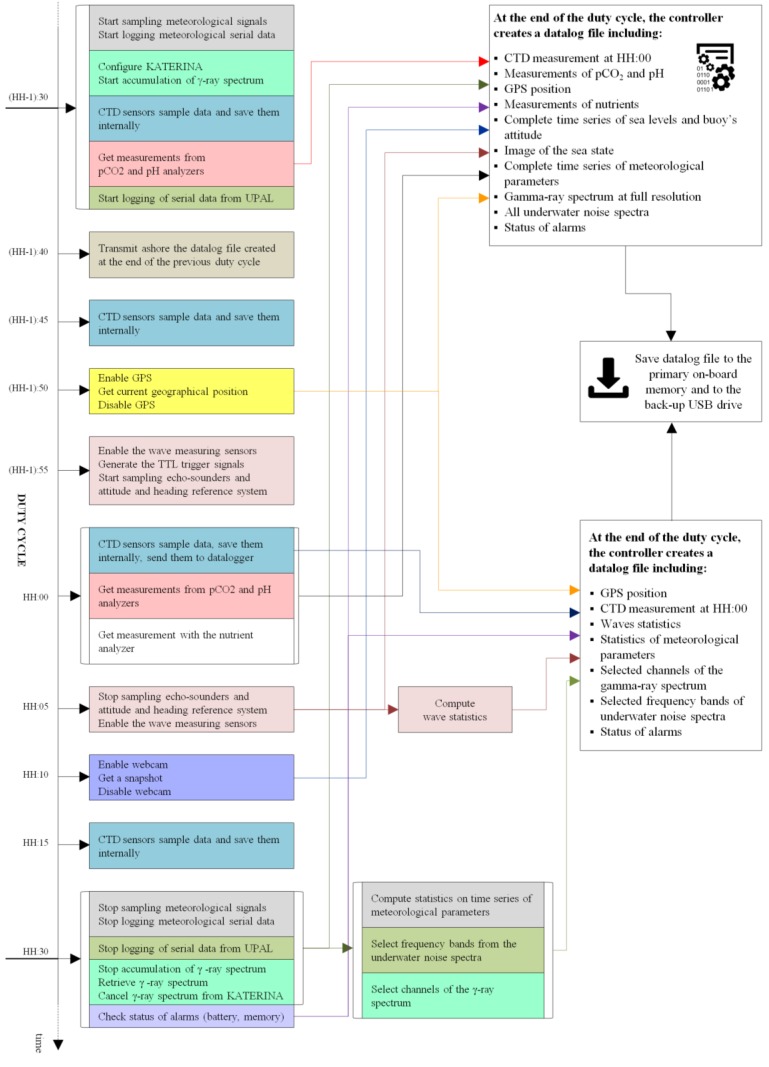
Sequence of processes implemented by the onboard real-time controller to integrate KATERINA II and UPAL in the data flow of the W1-M3A observatory. The duty cycle of one hour refers to a generic time instant HH:00. Reference times on the vertical temporal axis are not to scale.

In order to guarantee the full integration of the KATERINA II and UPAL sensors into the observation system a specific flow of actions has been defined to guarantee synchronization between spectroscopy data, acoustics samples and all other ancillary measurements based on the reference timing obtained by the GPS onboard the platform (Figure 3).

Specifically, the KATERINA II system was set up to acquire one spectrum per hour in sequential mode. At the end of a duty cycle, that is the time instant of enablement of a new acquisition period, the controller initiates the sampling of all meteorological instruments and sends the starting command to the KATERINA II. One hour later, the controller instantaneously interrupts the acquisition of the whole on-board instrumental payload to perform calculations of statistical analysis on meteorological observations and to retrieve the hourly cumulative gamma-ray spectrum. Subsequently, the controller deletes the data saved on the internal memory of the gamma-ray spectrometer and initiate the repetition of the whole acquisition process.

The time latency between the end of one acquisition period and the beginning of the next one is less than 10 s. As soon as a gamma-ray spectrum is retrieved, an automated routine (running in parallel with the acquisition), decodes the hexadecimal output of the sensor, translates the output into string, selects the values of the channels corresponding to ROIs of ^214^Pb, ^214^Bi, ^40^K, ^208^Tl and integrates them in the dataflow of the W1-M3A system to be transmitted ashore, whereas the full spectra is saved on the onboard compact flash memory.

Since UPAL uses an adaptive sampling strategy and does not allow a polling mode during operation, a listening subroutine was developed. As soon as a new message is made available by UPAL, it is transmitted to the main controller of the buoy through RS232. This information is then included in the main data stream of the buoy and transmitted ashore with a reference time for each acquisition.

As regards the physical installation, the KATERINA II unit was clamped to the main body of the observatory and close to the surface (at 6 m depth) in order to monitor the variations of ^222^Rn progenies concentration. Its two cables (one for the bidirectional communication and one for the power supply) were fixed along the body of the buoy up to the microcontroller, housed in the small laboratory on the top of the platform.

**Figure 4:**
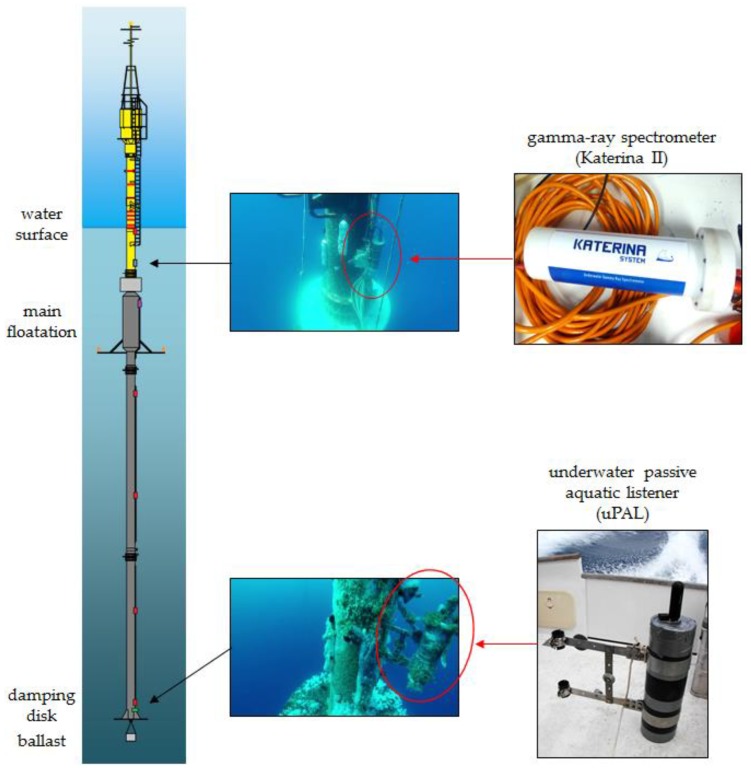
Sketch of the surface buoy of the W1-M3A observatory and images of the deployed sensors: the gamma-ray spectrometer at 6 m depth and the underwater passive aquatic listener close to the damping disk of the buoy at about 36 m depth.

Similarly, UPAL was clamped at the maximum available depth of the observatory (36 m) in order to provide acoustic information with a footprint on sea surface of about 100 m [31]. The depth of installation of UPAL was free from acoustic interferences due to the oceanographic payloads of the observatory. The cables for the bidirectional communication and the power supply were fixed in the same way as those of KATERINA II (Figure 4).

## 5. Experimental Results

The capability of the W1-M3A observing system to integrate underwater radioactivity and acoustic measurements into its already operational multidisciplinary payload was tested during Fall 2016 (from August to November).

The wake-up intervals of UPAL were set to one minute when rain was detected, two minutes in case of mammal vocalization and seven minutes for idle mode. The acoustic data were processed by UPAL using the classification and quantification algorithms described in [32] and, in post-processing, the retrieved estimates of rainfall were cumulated hourly to be comparable with the other measurements acquired during the experiment.

**Figure 5:**
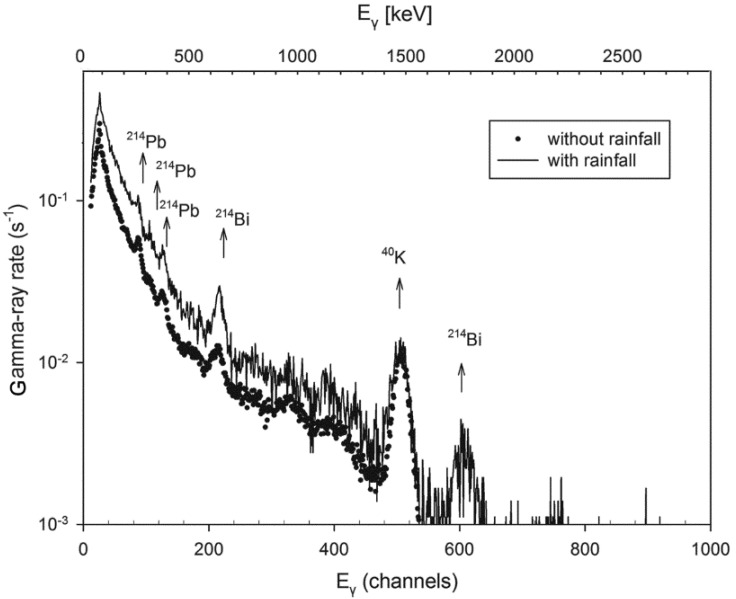
Gamma-ray spectra acquired by the KATERINA II system with and without rain. The gamma-ray rate is plotted versus channels (raw data, bottom axis) and keV (energy calibrated, top axis).

The KATERINA II sensor provided one spectrum every hour and two types of calculations were performed in an automated way: (a) gross gamma-ray rates were measured by summing the counts in the whole energy range (10–2700 keV) and dividing by the acquisition time in seconds (3600 s), and (b) counting rates in ROI obtained by summing the counts in the energy region of 351 keV for ^214^Pb, 609 and 1764 keV for ^214^Bi and dividing by the acquisition time. The quantification of the radioisotopes activity concentrations requires the analysis of the obtained spectra either manually using available software [33], or using dedicated automated algorithms [34]. Also, in order to obtain statistically acceptable results, given the short acquisition time (3600 s), grouping and summation of several spectra may be needed following appropriate analysis approaches [35]. Although quantification as well as association of activity concentrations with meteorological parameters are future tasks—out of the scope of the present work—for the sake of completeness, two typical gamma-ray spectra acquired during periods with and without rainfall are depicted in Figure 5. The first spectrum (depicted by dots) represents an indicative spectrum obtained during dry periods. The short acquisition time and the low activity concentration of radon progenies in the open sea, result to spectra of poor statistics, thus this spectrum was prepared by summing several hourly obtained spectra followed by appropriately time normalization. The spectrum depicted by the straight line was obtained during the storm on 10 of October 2016 with an acquisition time 3600 s. In the first one, the photo-peak corresponding to ^40^K dominates, as potassium generates the enhanced continuum of Compton background in the range 10–1460 keV. Slight photopeaks attributed to radon progenies can be observed in the energy regions of 352 and 609 keV (^214^Pb and ^214^Bi) attributed to background traces of lead and bismuth contained within the material of the station’s structural frame. After a rainfall event, several photopeaks attributed to radon progenies can be observed. More specifically, photopeaks of ^214^Pb are observed at the energies of 241, 270 and 351 keV and of ^214^Bi at the energies of 609 and 1764 keV.

**Figure 6:**
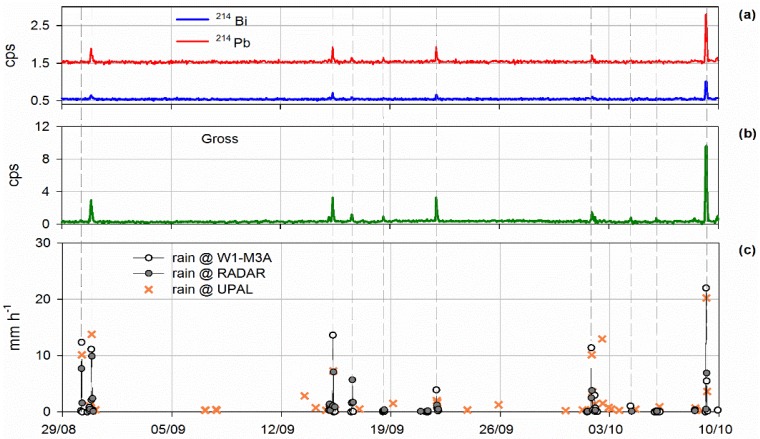
(**a**) Counting rate measurements of gamma-rays attributed to the radon progenies ^214^Pb and ^214^Bi; (**b**) Total detected gamma-rays; (**c**) Rain measured by the rain-gauge on the observatory, the weather radars and estimated by UPAL.

The results from the automated analysis in the range of 352–609 keV are depicted in Figure 6a along with the total gamma-ray results (Figure 6b). The precipitation events as identified by the rain-gauge installed on the observatory and the hourly cumulative values provided by two C-band, dual-polarized weather radars positioned on Mount Settepani (Liguria Region) and Bric della Croce (Piedmont Region) at 1386 m and 736 m above sea level, respectively, are shown in Figure 6c. Observations from weather radars were selected as the closest pixel to the buoy of the regional mosaic (6.2–11.2° E; 46.0–46.6° N) with a spatial resolution of 800 m × 800 m provided by the Environmental Protection Agency of Piedmont Region and Environmental Protection Agency of Liguria.

The same clear correlation can be observed between the quantitative estimates of cumulative rain provided by UPAL and the measurement of the in-situ rain gauge (Figure 6c): UPAL was able to detect rain in all hours in which reference measurements evidenced precipitation. The error related to its cumulative amount compared to the radar estimates is less than the one compared to the rain-gauge: this could be explained as UPAL gives an estimate of rainfall on a wide area, like radar does, whereas the rain-gauge on the buoy provides a point measurement.

In most cases, rainfall led to increases of the counting rates whereas some acoustic data variations are attributed to other sources. Combination of the results from radon progenies counting rates and from the processing of acoustic data identified five cases of intense precipitation events. Considering the total gamma-ray rate, additional precipitation events can be identified that are well detected by acoustic samples. However, there is at least one rainfall event identified by the other sensors and gauges without significant variation of radioactivity level. This could be explained as follows: radon is an inert gas and after its exhalation from the Earth’s crust is transported by wind and air movements; consequently, several meteorological parameters, e.g. wind speed and direction, pressure and temperature, should be taken into account as they contribute to radon concentration variation in the atmosphere before and during a rainfall event. 

On the other hand, acoustic data identify episodes when no rain was observed as rainfall events. It is worth noting that, analyzing the rainfall phenomena both with gross gamma-ray rate from KATERINA II and acoustic samples from UPAL, it is possible to identify correctly all the events, discarding any false alarm. In these cases, UPAL can provide very satisfactory approximation of rainfall amount in the area of the deployment. Further statistical analysis of the results is expected to reveal interesting new findings concerning rainfall and cloud characteristics (e.g., intensity, cloud high, age of raindrops) establishing radon progenies and acoustic observations as useful, alternative tracers of rainfall events.

## 6. Discussion and Conclusions

The integration of innovative sensors into existing observing systems significantly contributes to the increase of observational capacity of the platforms and to the provision of new insights for obtaining a more comprehensive picture of the sea state. To this aim, the simultaneous integration of two innovative sensors to monitor underwater radioactivity and ambient sound into the W1-M3A marine observing system was described and first results were presented.

In conventional observing platforms, deployment, setting and data management of new sensors is complicated because of the necessary integration with pre-existing scientific payload composed of heterogeneous sensors which operate separately. Furthermore, the installation and efficient long-term operation of underwater sensors on fixed-point observing systems demands compact instrumentation, easy to clamp, low-power consumption and minimum maintenance.

As regards the integration of the KATERINA II sensor into the W1-M3A observation system, the spectrum stability was guaranteed by using digital electronics modules adjusting the voltage output of the intense peak of ^40^K (at 1461 keV as measured due to the seawater constituents) in the middle of the spectrum (around 512 channel). Long term operation was ensured by the low power consumption of the system (~1 W) and the communication protocol was updated by programming the data-logger to operate via RS232 interface. Also, software functions were developed to achieve communication with the external controller of the observing system. The spectra were analyzed automatically for providing time series of the radon progenies and were transmitted in (near) real-time mode to the operational center.

As concerns the sound sensor upgrade, the UPAL sensor uses new low-consumption active filters with better cut-off frequencies, high internal data storage components (up to 128 Gb), and the versatility to be connected to any external power station. Also, for the purpose of integration new low power, low noise, high bandwidth, wide dynamic range combined microcomputer/ microprocessor and ADC, based on FPGA technology capable to accommodate the firmware and the processing smart algorithm were exploited. New solar panels and a wind generator were added to the power supply system of the W1-M3A, and the acquired measurements were included in the data stream of the system to be available ashore in near-real time.

The proposed multi-sensor integration can be easily replicated in other observing platforms for long-term monitoring purposes exploiting the capability to deploy the KATERINA II and UPAL sensors as a single instrumental package. In fact, the two innovative sensors can be installed through clamps on different types of infrastructures since the power supply can be either internal (battery pack) or external and both have the capability to communicate with standard protocols (RS232 and USB) in a two-way mode. The operation of both KATERINA II and UPAL can be fully re-configurable through specific commands (i.e., to modify the duration of the acquisition phase for the radioactivity spectrometer, to modify the wake–up intervals for the acoustic sensor, etc.) that have been implemented in the main controller of the hosting platform. The bidirectional communication has been also exploited to retrieve in near real-time a subset of the data acquired by the two sensors. Indeed, a subset of commands have been also coded in a configuration message which can be sent by users to the observatory using the satellite communication system at the end of the periodic data transfer to the receiving station ashore. In this way, it is possible to modify the operation of the two sensors to better investigate on demand particular events, for example by increasing the rate of collection and processing of sound clips or focusing on particular bands of energy.

The graphical user interface (GUI) of each instrument is immediate to use even by non-experts and allows one to easily program and set-up the sampling options for any deployment or experiment. Furthermore, in the case that the developed software and hardware tools are embedded on other existing infrastructures able to provide data to international repositories, it will support the scientific community on issues related to underwater radioactivity and sound, since in-situ measurements of these parameters are very scarce up to now.

Preliminary analysis of the first results of the combined use of continuous monitoring of radon progenies near the sea surface and of underwater ambient sound identified five cases of intense precipitation events. These data in combination with ancillary meteo-oceanographic parameters (wind speed and direction, seawater salinity, etc.) may provide an interesting multidisciplinary alternative for detecting and investigating precipitation characteristics (precipitation intensity, cloud heights, etc.) in remote oceanic areas. This alternative approach may contribute to validating satellite precipitation observations, while still providing valuable information about the local ecological status (radioactivity and noise budget levels, marine mammal identification, etc.) of the studied marine area.

In conclusion, the integration of radioactivity and sound sensors in stationary observation systems, based on universally applicable methods, provides a modern and multidisciplinary scientific approach of gathering important new information to better understand the functionality of the marine environment.

## Supplementary Materials

The meteorological data used for the analysis are freely available from the portal of the Copernicus Marine Environment Monitoring Service (http://marine.copernicus.eu) and from the web site of the Regional Environmental Protection Agency of Liguria (https://www.arpal.gov.it). Acoustic and radon data are available at the following address: http://www.w1m3a.cnr.it/OI1/modules/site_pages/fixo3_TNA.php.
